# Towards the Structural Health Monitoring of Bridges Using Wireless Sensor Networks: A Systematic Study

**DOI:** 10.3390/s23208468

**Published:** 2023-10-14

**Authors:** Omar S. Sonbul, Muhammad Rashid

**Affiliations:** Computer Engineering Department, Umm Al Qura University, Makkah 21955, Saudi Arabia; mfelahi@uqu.edu.sa

**Keywords:** structural health monitoring, bridges, WSN, energy harvesting, systematic literature review

## Abstract

To perform a comprehensive assessment of important infrastructures (like bridges), the process of structural health monitoring (SHM) is employed. The development and implementation of SHM systems are generally based on wireless sensor networks (WSN) platforms. However, most of the WSN platforms are battery-powered, and therefore, have a limited battery lifetime. The power constraint is generally addressed by applying energy harvesting (EH) technologies. As a result, there exists a plethora of WSN platforms and EH techniques. The employment of a particular platform and technique are important factors during the development and implementation of SHM systems and depend upon various operating conditions. Therefore, there is a need to perform a systematic literature review (SLR) for WSN platforms and EH techniques in the context of SHM for bridges. Although state-of-the-art review articles present multiple angles of the field, there is a lack of an SLR presenting an in-depth comparative study of different WSN platforms and EH techniques. Moreover, a systematic analysis is also needed for the exploration of other design considerations such as inspection scale (global/local), response type (static/dynamic), and types of sensors. As a result, this SLR selects 46 articles (during 2007–2023), related to EH techniques and WSN platforms in SHM for bridges. The selected articles are classified into three groups: WSN platforms, energy harvesting techniques, and a combination of both. Subsequently, a comparative analysis of WSN platforms and EH techniques is made. Furthermore, the selected articles (total = 46) are also explored in terms of sensor type, inspection scale, and response type. As a result, 17 different sensor types are identified. This research is significant as it may facilitate the various stakeholders of the domain during the selection of appropriate WSN platforms, EH techniques, and related design issues.

## 1. Introduction 

The development of an efficient infrastructure is getting more and more important with an enormous increase in population. The term infrastructure includes different types of systems, tools, processes, equipment, and services [1,2,3,4,5]. Among others, bridges are one of the important infrastructures from the cost and safety point of view. However, the strength of bridges becomes progressively worse due to several factors. These deteriorating factors include but are not limited to cyclic loads, creep, and corrosion. Therefore, continuous health monitoring of the bridges and appropriate maintenance procedures are essential. In other words, a damage deification process must be employed [6,7,8,9,10]. 

The damage identification in bridges has been performed manually for several years. Nevertheless, there are several drawbacks to manual monitoring such as dependency on the inspection team, increased time, and inefficacy in finding the flaw growth stages [11]. Consequently, the automation of the damage identification process is critical using an appropriate structural health monitoring (SHM) system. A typical SHM system is based on various sensors placed at different locations of the structure. The data is received from these sensors and analyzed accordingly [12,13,14]. The SHM systems for bridges have been previously implemented using sensor networks with wires. However, the great advancements in wireless technology have given birth to wireless sensor networks (WSNs). The installation and maintenance costs of WSN platforms are much less than wired sensor networks. Therefore, WSN platforms have been frequently employed for the SHM of bridges [15,16,17,18,19,20]. 

The WSNs are deployed during the SHM process, either with global or local damage identification techniques [21]. Global techniques are used to identify the presence of damage and evaluate the health of the complete structure. On the other hand, the local techniques help in finding the damage at a specific place or focusing on a particular parameter in the structure [22]. In other words, global measurement techniques are suitable for detecting large faults, whereas local measurement techniques are generally employed for detecting all those damages that cannot be measured using global measurement techniques [23]. 

To carry out any damage identification technique (local as well as global), there exist many damage detection methods. In these damage detection methods, an excitation is provided through a source. Subsequently, the response of the structure is monitored accordingly. The damage detection methods are classified into two main categories: static and dynamic. Examples of static response monitoring are strain and stress. Similarly, examples of dynamic response monitoring are frequencies, mode shapes, and modal damping. It has been observed that the measurement of static responses is more straightforward as compared to dynamic measurements [21]. Nevertheless, static responses are relatively less sensitive to changes resulting from the damage. Consequently, the monitoring of dynamic responses is generally preferred for the detection of abrupt as well as gradual changes. The limitations of dynamic monitoring are the collection of correct data due to certain environmental and operational factors. 

Finally, most of the WSN systems for the monitoring of the structural health of the bridges are battery-powered. As a result, a major constraint in this context is the limited lifetime of the battery. Therefore, periodical high-cost battery maintenance is needed to overcome this major constraint for WSN sensors. In other words, the constraint of the limited lifetime of the battery is generally addressed by applying energy harvesting technologies. In an energy harvesting (EH) process, the power is collected from ambient energy sources. There are different types of ambient energy sources including solar, vibrations, wind, thermal, and radiofrequency (RF). The selection of the most appropriate energy harvesting technique for a specific SHM system depends on different factors such as the environmental conditions, the type of bridge, and the availability of the source for radio frequency signals [24]. 

### 1.1. Motivation for the SLR

As described earlier, the WSNs introduce a viable option platform in the field of SHM for bridges. Various WSN platforms have been deployed for the SHM of bridges with multiple types of sensors. These SHM systems can be classified according to the inspection scale (global/local) and the response type (static/dynamic). Furthermore, energy harvesting techniques including solar, thermal, wind, FR, and vibration-based are deployed to overcome the major constraints of the battery-powered WSN systems. Therefore, a review based on a systematic process is needed to evaluate state-of-the-art WSNs for SHM of bridges.

### 1.2. Limitations of Existing Reviews 

Table 1 describes the salient features of existing review articles along with their limitations on WSN platforms and EH techniques [24,25,26,27,28,29,30,31]. The table reveals that state-of-the-art review articles on this topic elaborate on various issues and challenges. Nevertheless, a comprehensive review observing a systematic process on WSN platforms and EH techniques for the SHM of bridges is lacking. In addition, the analysis of some other design parameters such as sensor types, inspection scale (global/local), and response type (static/dynamic) is also essential. 

### 1.3. Contributions

The limitations of state-of-the-art review articles, highlighted in Table 1, have been rectified by performing a systematic literature review (SLR). Particularly, the SLR has explored the answers to the following four research questions:

**Research question 1**: What are the most important WSN platforms and EH techniques, reported in the research articles from 2007 to 2023, that have been utilized for the SHM of bridges?

**Research question 2**: Which of the EH techniques is more effective for the SHM of bridges, based on the research articles from 2007 to 2023?

**Research question 3**: What are the most important sensor types that have been employed for the SHM of bridges, based on the research articles from 2007 to 2023? 

**Research question 4**: Which of the system inspection scale techniques and response types are most frequently utilized in the process of SHM in bridges, based on the research articles from 2007 to 2023. 

### 1.4. Overview of the Conducted SLR

**Figure 1:**
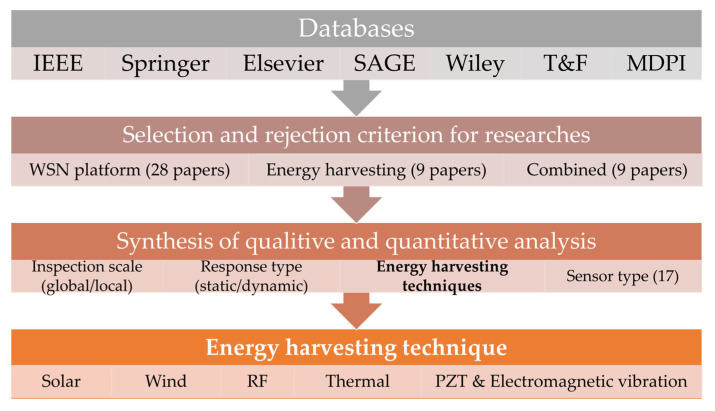
Overview of the research.

Figure 1 depicts the top-level view of the entire SLR process conducted in this study. Using seven scientific databases (i.e., IEEE, Springer, Elsevier, SAGE, Wiley, MDPI, and Taylor and Francis), the research articles are scrutinized using a well-defined criterion. Section 2 elaborates on the methodology (criterion) used for the selection of 46 research articles. These research articles are then divided into three categories: WSN studies (28 research articles), energy harvesting studies (9 research articles), and combined studies (9 research articles). Section 3 explores the selected research articles according to various parameters, including sensor type (17 types), inspection scale (local/global), and response type (dynamic/static). While Section 3 overviews the WSN platforms and EH techniques, a comprehensive comparison is provided in Section 4. The energy harvesting techniques include solar, wind, thermal, radiofrequency (RF), and vibration-based (electromagnetic and piezoelectric material (PZT)). Section 5 provides answers to the formulated research questions. Section 6 provides a discussion of the achieved results along with the limitations of the employed process. The concluding remarks are given in Section 7.

## 2. Research Methodology

The SLR process, described in [32], has been employed to execute this research. The following subsections provide the corresponding details on six different phases of the process. 

### 2.1. Background on Categories

Three categories have been defined to classify the selected research articles. The purpose is to enhance the correctness of the responses to our formulated research questions in the introductory part of this article. The essential background of the three categories is provided in the following subsections.

#### 2.1.1. WSN Platforms

In a typical WSN platform, employed for the SHM of bridges, sensors are placed at multiple places of the bridge. The deployed sensors, which are scattered throughout the structure, collect information about their environments. The collected information includes but is not limited to acceleration, ambient vibration, load, and stress at higher sampling frequencies (upwards of 100 Hz). Similarly, the size of collected data in the SHM application is another issue. Therefore, the network design using a WSN platform poses serious challenges for the SHM systems. In addition to this, data aggregation and processing are necessary for the detection and localization of structural damage. The data aggregation and processing activities may occur at multiple places depending upon the employed network topology [27]. The research articles in which a WSN platform is deployed for the SHM of bridges are included in this category. 

#### 2.1.2. Energy Harvesting Techniques 

Battery-operated WSN platforms face some serious power consumption issues. The life span of a battery-powered WSN platform is not enough to operate for longer durations. To address this drawback, certain maintenance operations are carried out on a regular basis. An example of these maintenance operations is the recharging of the battery-operated system. In some cases, the batteries are entirely replaced. As a result, the operational cost of the systems has increased significantly. A promising alternative to the costly manual maintenance procedure (such as recharging or replacing batteries) is energy harvesting. During this phenomenon, the energy is taken from an ambient source. Typical examples of ambient energy sources are sunlight, wind, vibration, sound, heat, and radio frequency (RF) [26]. The research articles in which an energy harvesting technique is deployed for the SHM process are included in this category. The energy harvesting techniques considered in this SLR include solar, wind, thermal, RF, electromagnetic vibration-based, and PZT vibration-based. 

#### 2.1.3. Combined

The research articles in which a WSN platform and an energy harvesting technique are deployed at the same time for the SHM process of bridges are included in the combined category. These research articles provide a complete system that can be used as a standalone platform to overcome the constraints of battery-operated systems. These platforms can work smoothly and independently for weeks without any human intervention. Consequently, this will increase the reliability and maintainability of the proposed system. Furthermore, this leads to low installation and maintenance costs. 

### 2.2. Review Protocol Development

After the definition of categories, a protocol is developed. The developed protocol is used to carry out the entire SLR process [32]. It includes the following steps: the development of a selection and rejection criterion, the description of the search process, the quality assessment of the selected research studies, data extraction, and the synthesis of the extracted data. The corresponding information on each step of the review protocol is given in the following subsections. 

#### 2.2.1. Selection and Rejection Criterion

The research article is selected according to the following parameters:

*Relevancy*: The primary concern in the selection of a research article is its relevance to the target research area and must assist in responding to formulated research questions. 

*2007–2023*: The publication date for the selected research article is from 2007 to 2023. 

*Publisher*: The research article is published in one of the seven famous databases, i.e., IEEE, SPRINGER. ELSEVIER, SAGE, Wiley, MDPI, and Taylor & Francis.

*Crucial effects*: The selected research article must have crucial positive effects regarding the deployment of WSN platforms or EH techniques for the SHM of bridges. A new algorithm or technique should be reported in the selected article. 

*Clear outcomes*: The selected research article must have clear outcomes. The major steps in the proposal are clear and an appropriate validation mechanism must be given. 

*Repetition*: There are several research articles with similar context and findings. It is too hard to include all such research articles in the SLR. As a result, only a single article is included as a representative of other articles. The pioneer paper is selected unless the new paper presents a new methodology or algorithm.

#### 2.2.2. Search Process

The target databases for our search process are shown in Table 2. Therefore, we have used different keywords in these databases. The employed time filter is “*2007–2023*”. The achieved results with the AND operator may not confirm the relevance of the research context. Consequently, the OR operator has been used. It allows us to obtain relatively more sound and concrete search results. Nevertheless, the OR operator generates an enormous number of results. The scanning of this enormous amount of data is a daunting task. Therefore, two additional filters have been employed. These filters are “content type = article”, and “subject area = Engineering”. 

**Figure 2:**
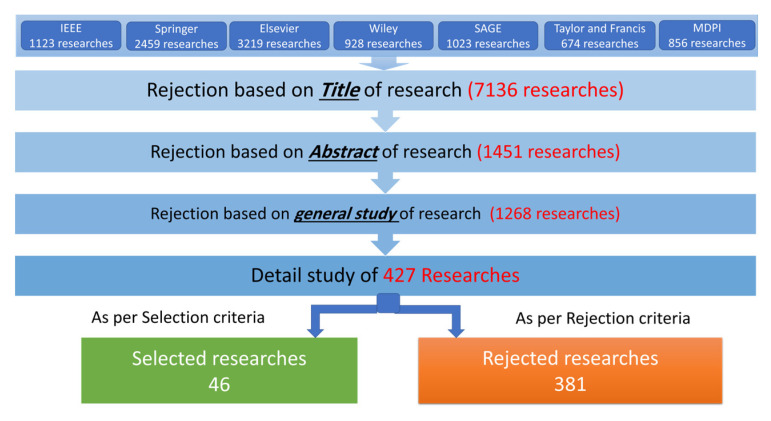
The flow of the employed search process.

Figure 2 elaborates various steps in the search process. Different search terms were used in the target databases. Consequently, we analyzed approximately 10,282 search results. In the next step, 7136 research studies were filtered out by observing their *Title.* Similarly, 1451 research articles were filtered out by observing their *Abstracts*. Then, we performed a general study of 1695 articles. During this general study, the coherency among different sections as well as the formulated research questions (RQ1 to RQ4) were checked. Based on our general study, we discarded 1268 articles that did not meet the selection and rejection criterion. As a result, 427 articles were selected for a comprehensive evaluation. Based on the comprehensive evaluation (termed as the “detailed study” in Figure 2), we were able to filter 381 articles. In the last step, 46 research articles were finalized.

#### 2.2.3. Quality Evaluation

**Figure 3:**
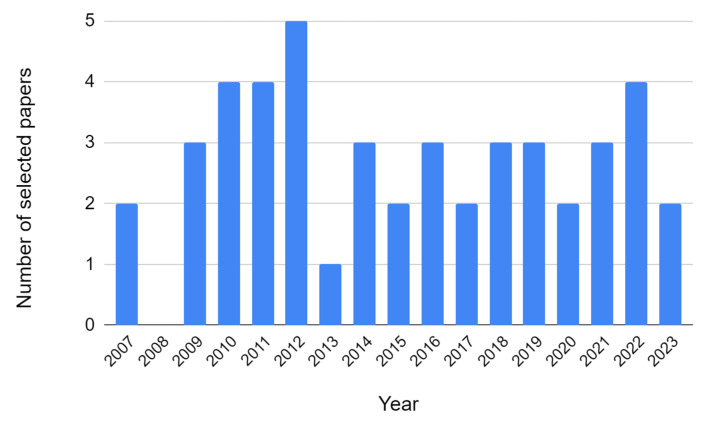
Statistics of research articles according to the publication year.

The following criteria were used to evaluate each selected research:

(1) A rigorous validation of the selected research must have been conducted.
(2) The research must provide the implementation details of the corresponding system.
(3) In order to preserve originality, seven renowned scientific databases, i.e., IEEE, SPRINGER, ELSEVIER, SAGE, Wiley, MDPI, and Taylor & Francis were targeted.
(4) To obtain the most recent results on the applications of the WSN platforms and energy harvesting techniques, we included research articles from 2007 to 2023 as shown in Figure 3.
(5) There are some high-quality conference papers in the field; therefore, we included 6 conference papers and 40 journal articles.

## 3. Results

This section presents the results obtained from the selected studies [33,34,35,36,37,38,39,40,41,42,43,44,45,46,47,48,49,50,51,52,53,54,55,56,57,58,59,60,61,62,63,64,65,66,67,68,69,70,71,72,73,74,75,76,77,78]. The results suggest that the pioneer efforts in the context of these four parameters (sensor types, energy harvesting techniques, damage inspection techniques, and damage detection methods) have been made during the first two decades of the twenty-first century. Therefore, most of the selected studies for this SLR are within this period. It has also been observed that most of the new articles in the domain of the deployment of WSN (SHM applications) focus on different aspects, such as communication protocol, time synchronization, feature extraction, and pattern recognition. These parameters such as communication protocol, time synchronization, feature extraction, and pattern recognition do not lie within the scope of this article.

Data extraction and synthesis, as shown in Table 3, were performed to get the answers to our research questions. An overview of the classification of selected studies into three predefined categories (WSN platforms, energy harvesting techniques, and a combination of both) is provided (Section 3.1). In addition to the three main categories, there are three more design factors. These design factors/parameters are sensor utilization (Section 3.2), system inspection scale (Section 3.3), and response type (Section 3.4).

### 3.1. Overview of Results: Classification of Selected Studies into Defined Categories

Section 2 of this article has defined three categories. WSN platforms, energy harvesting techniques, and a combination of both (WSN platforms as well as energy harvesting techniques). Consequently, 28 studies have been selected in the first category. Similarly, nine studies have been selected in the second category. Finally, the combined category includes nine studies. The number of selected studies in the first category is higher compared to the other categories. This is because most of the WSN platform investigation studies that applied SHM for bridges are battery-operated and do not apply energy harvesting techniques.

A comprehensive analysis of different WSN platforms is presented in Section 4.1 of this SLR. The parameters of the comparison study include data acquisition specifications, embedded computing specifications, and wireless channel specifications. It can be observed from Table 4 that the frequently employed EH techniques are the vibration-based (11 studies) and the solar (6 studies) approaches. A comprehensive analysis of different EH techniques is presented in Section 4.2 of this SLR. The parameters of the comparison study include output voltage and power, rectification specifications, and integration specifications.

### 3.2. Sensors’ Utilization

The use of a particular sensor type is a critical decision during the development and implementation of the SHM process. Similarly, the performance attributes/factors/parameters for which the data are collected is another important decision. The entire network design depends upon the selection of sensor type and performance attributes. Based on these decisions, the routing protocol is targeted. Similarly, the algorithms for damage detection and localization are entirely based on the type of sensors and the corresponding performance attributes.

It can be observed from Table 5 that the most important sensors in the selected research articles are accelerometers (27 studies), temperature sensors (14 studies), and strain gauges (11 studies). The accelerometer is used to indirectly measure the stiffness, pylons, and hanger cables in SHM applications using vibration measurement. It is frequently utilized due to its low cost and ease of use. The temperature is commonly used in SHM applications for compensation and estimation purposes of environmental factors. Table 5 shows that some types of sensors have been utilized only in one selected article. This utilization is for a specific purpose. It includes acoustic emission (AE) sensors, wind gauge sensors, RFID sensors, infrared sensors, light sensors, sound sensors, and polyvinylidene fluoride (PVDF) sensors.

The AE sensors utilize stress waves. These stress waves are generated by the immediate adjustment of internal stress. Some possible causes for this immediate adjustment are the growth of fatigue cracks and dislocation movements. In other words, the primary reasons for the stress waves are concerned with the damage. Therefore, the assessment of acoustic emissions can be linked with the failure of the material and can be sensed by using an AE sensor [79]. For example, Ledeczi et al. [39] proposed a real-time system for monitoring the active fatigue cracks in bridges using the acoustic emission sensor. The system is capable of the detection and accurate localization of fatigue cracks with high accuracy.

The PVDF piezoelectric-film sensors (abbreviated as PVDF sensors) are based on the piezoelectric effect. The piezoelectric effect is the capacity of different materials to produce an electric charge across its boundaries. The electric charge is produced when mechanical stress is produced [80]. For example, Junhee et al. [64] proposed an effective strategy using accelerometers and PVDF sensors. The accelerometers are placed in the vertical direction. For recording the time, five PVDF sensors are bonded to the top surface of the deck at the abutments and piers. The PVDF tactile sensors are designed to generate voltage when the vehicle applies vertical pressure.

### 3.3. Inspection Scale Investigations (Global Techniques Versus Local Techniques)

It is mentioned in Section 1 that the employed techniques in the damage identification process are classified into global techniques or systems and local techniques or systems. In global systems, a given load is applied. The associated response with the given load is assessed accordingly. Acceleration and velocity are the commonly used parameters in global systems. In contrast, the measurement of a response to a given load, which can only be assessed in a particular component/portion, is deployed in local systems. Typically, measured parameters in local techniques are strain, crack, and tension forces.

As can be seen in Table 6, the global systems are frequently utilized (31 research studies). On the other hand, the local methods are deployed for simple structures only. Although the local methods identify the location of the faulty component, a tremendous amount of time and cost are required. Therefore, global techniques are generally preferred in complicated structures. Global techniques provide useful data by utilizing vibration characteristics.

In addition to the use of global and local techniques in isolation, a combination of global and local techniques is also used. The global approach is first used for damage detection. Subsequently, the local approach is used for damage assessment and localization. For example, Musiani et al. [68] presented a solution with a combination of local and global methods. It consists of two layers of sensors, including RFID and PZT sensors. The RFID coupled with the PZT sensor is used to detect the peak strain from the RF signal provided by unmanned aerial vehicle (UAV). If the values of peak strain are not within the limits, the activation of corresponding nodes takes place, and the damage is detected accordingly.

### 3.4. Response Type (Static Methods Versus Dynamic Methods)

Both global and local techniques employ certain methods for the SHM of bridges. These methods are classified according to the response type. The two types of responses are dynamic (such as frequencies, mode shapes, and modal damping) and static (strain or stress) systems. The inaccuracies in static systems are almost negligible as compared to dynamic responses. Moreover, static measurements only require the structure’s stiffness matrix. On the other hand, the dynamic matrix requires stiffness, mass, and damping matrices. For this reason, static response-type methods generally have simpler equations. The static properties are primarily related to the steady state values of the measuring equipment. In other words, transient values are not the main concern in static techniques. On the other hand, the dynamic properties are mainly concerned with transient values.

As can be seen in Table 7, the dynamic response system is generally preferred over the static method (18 studies). Moreover, both dynamic and static response analyses have been deployed in 15 selected studies. Here, the static response measurement is mainly used to compensate for the environmental factors (temperature and humidity). For example, Mascarenas et al. [71] proposed a static response system that consists of WSN nodes. The sensor nodes utilize peak strain sensors to monitor the maximum displacement. The maximum displacement is a result of some tangible load input. A typical example of this significant loading event is an earthquake. The measurement of maximum displacement may result in significant life safety and economic benefits.

## 4. WSN Platforms and Energy Harvesting Techniques Investigations

Section 3 describes the classification from the selected articles [33,34,35,36,37,38,39,40,41,42,43,44,45,46,47,48,49,50,51,52,53,54,55,56,57,58,59,60,61,62,63,64,65,66,67,68,69,70,71,72,73,74,75,76,77,78] in terms of various parameters. However, an in-depth comparative study of WSN platforms as well as energy harvesting techniques is the primary objective of this article. Therefore, this section compares WSN platforms and energy harvesting techniques in terms of various performance attributes. Section 4.1 analyzes various WSN platforms while the energy harvesting techniques are investigated in Section 4.2.

### 4.1. WSN Platforms

The three main components of a typical WSN platform are data acquisition, embedded computing, and wireless channel systems. The specifications of these components affect the reliability as well as the accuracy of the platform. There are 37 articles (out of 46) that have deployed the WSN platforms (28 studies in the first category and 9 studies in the combined category).

Table 8 presents an overview of the selected studies in the context of WSN platforms. The second column identifies the name of the prototype for the corresponding WSN platform. The data acquisition specifications are presented in the third column. Similarly, the specifications of the embedded processor are expressed in the fourth column. Finally, the last column presents the specifications of the wireless channel in the data communication process. Regarding the specifications of data acquisition, most of the platforms deploy ADC with 12-bit resolution. It is adequate to digitize the input analog signals from the sensors with high accuracy. In addition, most of the platforms have eight channels. As far as the embedded computing specifications are concerned, the ATmega128 (64 KB SRAM + 48 KB Flash) and the TI MSP430F1611 processors (10 KB SRAM + 48 KB Flash) are the frequently used microcontrollers. These processors have adequate size of data and program memory for SHM applications. Finally, regarding the specifications of the wireless channel systems, the CC2420 is the most used RF transceiver that supports data rates of up to 250 kbps. The Zigbee technology is the most frequently used wireless protocol in the selected WSN platforms.

Table 9 presents a summary of the accuracy of WSN systems. It is related to the measurement of different quantities such as acceleration and strain, acoustic emission, and impedance. As can be seen in Table 9, only 13 studies (out of 37 selected studies) are included. This is because the accuracy of the proposed system is not mentioned in the other 24 selected studies. The probable reason for the lack of this information is that these studies focused on the development and assessment of the WSN systems in terms of data transmission and acquisition. It can be seen in Table 9 that most of the proposed WSN systems exhibit reasonable accuracy in measuring different quantities as compared to wired or commercial systems. For example, Komarizadehasl et al. [62] applied the Modal Assurance Criterion (MAC) to compare the mode shapes of the analytical analysis and the experimental study performed by the proposed WSN system. The results of the comparison using MAC were within the range of 0.93 to 1.00. In addition, the study compares the eigenfrequencies measured by the proposed system with those of commercial acceleration accelerometers, which showed a maximum difference of 0.87 Hz with a difference of 3.3%.

### 4.2. Energy Harvesting Techniques

The major performance parameters for the analytical comparison between various energy harvesting systems include (1) the output voltage and power, (2) the rectification process, and (3) the integration process. There is a wide range of ambition energy sources such as solar, wind, and RF. The selection of the most appropriate energy harvesting technique for a specific SHM system depends on different factors such as the environmental conditions, the type of the bridge, and the availability of the source for radio frequency signals. Energy harvesting techniques have been employed in 18 research studies (9 in the second category and 9 in the combined category). The selected studies cover the following energy harvesting techniques: vibration, solar, wind, radio frequency (RF), and thermal. It is important to note that the eleven selected studies for vibration energy harvesting techniques are further categorized as six studies for electromagnetic-based vibration energy harvesting systems and five studies for piezoelectric material-based vibration energy harvesting systems. There are four research studies that combine two different energy harvesting techniques. For example, the solar and wind techniques have been jointly deployed in two studies [38,69]. Similarly, electromagnetic vibration and wind techniques are simultaneously applied in [70]. Finally, the PZT vibration and the thermal techniques are combined in [73]. The following sub-sections provide a comprehensive analytical comparison of different energy harvesting techniques in terms of various performance attributes.

#### 4.2.1. Vibration Energy Harvesting Systems

There are mainly two types of mechanical vibration energy harvesting: (1) piezoelectric material (PZT) vibrations and (2) electromagnetic generator vibrations. Piezoelectric energy is produced by the mechanical rotation or vibration of piezoelectric patches. The electromagnetic vibration energy is generated by the relative movement of the magnet and coil. A mechanical vibration energy harvester is usually attached to ambient vibrating or moving objects. Typical examples of these moving objects are motors, machines, vehicles, and even human bodies. This is regarded as a very efficient approach to harvesting energy [24].

Table 10 and Table 11 summarize the electromagnetic-based and PZT vibration energy harvesting techniques. The second column depicts the peak output voltage. The total volume of the electromagnetic circuit is presented in the third column. The resonant frequency of the electromagnetic circuit is expressed in the fourth column. The fifth column presents the minimum value of acceleration (peak/RMS) for the conversion process. The generated output power (peak/average) of the conversion process is presented in the sixth column. The seventh and eighth columns express the rectification and integration processes. As can be seen in Table 10, the output power is directly proportional to the volume of the electromagnetic circuit used in the systems. For example, the system proposed by Yang et al. [45] delivers the maximum output peak power and average power of 2229 µW and 1147 µW, respectively with an electromagnetic circuit volume of 686.9 cm^3^.

The second column in Table 11 identifies the output voltage. The resonant frequency of the PZT circuit is expressed in the third column. The fourth column presents the generated output power of the conversion process. The fifth and sixth columns express the rectification and integration processes. As can be seen in Table 11, the AC-DC converter is the most used rectification technique to generate the DC voltage needed to recharge the integration capacitors.

#### 4.2.2. Solar Energy Harvesting Systems

Solar energy is a traditional ambient energy source that has already been widely investigated for its abundance and renewability. Solar energy has the highest power density among all the ambient energy sources. Twelve Photovoltaic (PV) cells are the key components of solar energy harvesters that generate electricity from the ambient sunlight [24]. Table 12 presents a summary of the solar energy harvesting techniques. The second column identifies the output voltage. The size of the photovoltaic modules (PV) is shown in the third column. The fourth column presents the performance of the conversion process in terms of the maximum output power and the conversion efficiency. The fifth and sixth columns express the rectification and integration processes. As can be seen in Table 12, the output power and voltage are directly proportional to the size of the PV module used in the systems. For example, Pękosławski et al. [69] proposed a solar energy harvesting system with maximum output power and output voltage of 28.4.4 W and 29 V, respectively, which are generated from a PV module with a size of 140 cm^2^, whereas the system proposed by Huynh et al. [78] is capable of generating only 3 W and 3.7 V with a 78 cm^2^ PV module.

#### 4.2.3. Wind Energy Harvesting Systems

Wind is the second most widely used renewable energy source for generating large-scale power. Large-scale wind power generation technologies have been well-developed and studied for many years. However, research on wind energy harvesting in a small-scale area, specifically for SHM systems, has only emerged in recent years. Wind energy can be accessed during the day and at night, and even under rainy and cloudy conditions as compared to solar energy. Since many bridges are in windy regions, some researchers have paid more attention to wind energy harvesting and regard it as a feasible energy source for wireless sensor nodes in SHM applications [81].

Table 13 presents an overview of the selected research studies for wind energy harvesting techniques. The second column shows the output voltage. The diameter of the wind turbine is expressed in the third column. The fourth column presents the performance of the conversion process in terms of maximum output power and conversion efficiency. The fifth and sixth columns express the rectification and the integration processes, respectively. As can be seen in Table 13, the output power and voltage are directly proportional to the diameter of the wind turbine used in the systems. For example, Pękosławski et al. [69] proposed a wind energy harvesting system with maximum output power and an output voltage of 22.4 W and 29 V, respectively. A wind turbine with a diameter of 1.14 m has been used during this process. On the other hand, the system proposed by Boyle et al. [38] is capable of generating only 10 mW and 5 V with a 6.3 cm wind turbine.

#### 4.2.4. RF Energy Harvesting Systems

The RF signals (from 3000 Hz and 300 GHz) signals are generated from millions of radio stations. Based on these RF signals, the RF energy harvesting process is defined as the phenomenon of taking energy from some ambient RF sources. Typical examples of ambient RF sources are MF (AM Radio, 526.5–1705 KHz); FM (87.5–108 MHz); TV (41–250 MHz, 470–950 MHz); GSM (850/1900 or 900/1800 MHz); CDMA, 3G, 4G, and ISM (industrial scientific medical, 2400 MHz); and Wi-Fi (2.45/5.0 GHz) [82]. Compared with the ambient energy sources mentioned above, RF energy is independent of environmental conditions, including weather, climate, and temperature. These advantages make it an attractive choice as an ambient energy source for powering wireless sensor nodes for SHM applications.

Table 14 presents an overview of the selected research studies for RF energy harvesting techniques. The second column identifies the output voltage. The sensitivity of the RF harvester is expressed in the third column. The fourth column presents the performance of the conversion process in terms of maximum output power and conversion efficiency. The fifth and sixth columns express the rectification and the integration processes, respectively. There is only one selected study in this SLR that has deployed the RF energy harvesting technique. Probably, this is the only work in this category that meets the selection and rejection criterion of this SLR.

#### 4.2.5. Thermal Energy Harvesting Systems

In this case, the ambient energy is extracted using thermal gradients. For this purpose, thermoelectric generators (TEGs) are utilized. They transform thermal gradients into electricity. During the transformation process, the Seebeck effect is used. When the Tegs are compared with the vibration based EH systems, it can be noticed that the former have no kinetic components [83]. Table 15 presents an overview of the thermal energy harvesting techniques. The second column identifies the output voltage. The maximum differential temperature is expressed in the third column. The fourth column presents the performance of the conversion process in terms of maximum output power and conversion efficiency. The fifth and the sixth columns express the rectification and the integration processes, respectively. There is only one selected study in this SLR that deployed the thermal energy harvesting technique. It shows that this technique is not widely deployed. This is due to the relatively low performance of TEGs for minor variations in temperature.

## 5. Responses to Formulated Research Questions

**Research question 1**: What are the most important WSN platforms and EH techniques, reported in the research articles from 2007 to 2023, that have been utilized for the SHM of bridges?

**Answer:** A total of 46 articles (from 2007 to 2023) have been identified using a well-defined systematic process, mentioned in Section 2 of this article. The selected articles are arranged into three groups.

Twenty-eight articles have been included in the WSN group (Section 4.1);

Nine articles have been included in the energy harvesting group (Section 4.2);

Nine articles have been included in the combined group.

**Research question** 2: Which of the EH techniques is more effective for the SHM of bridges, based on the research articles from 2007 to 2023?

**Answer:** The vibration-based (11 studies) and solar (6 studies) methods are the most used techniques. The vibration-based energy harvesting techniques are classified into electromagnetic and PZT methods. Further details are available in Table 10, Table 11 and Table 12**.**

**Research question 3**: What are the most important sensor types that have been employed for the SHM of bridges, based on the research articles from 2007 to 2023?

**Answer:** The most frequently used deployed sensors are accelerometers, strain gauges, and temperature sensors. The accelerometer is widely used to assess the dynamic response of the bridge structure, whereas the temperature sensors and strain gauges are deployed to assess the static response of the bridge structure. Further details are available in Table 5. There are other sensors that have been deployed in the SHM for bridges such as humidity, PZT, peak displacement, and ultrasonic sensors.

**Research question 4**: Which of the system inspection scale techniques and response types are most frequently utilized in the process of SHM in bridges, based on the research articles from 2007 to 2023?

**Answer:** The global scale investigations are the most used techniques in the SHM for bridges (31 studies out of 46), as shown in Table 6. Only five selected studies deploy the local scale investigation techniques. In addition, the dynamic response analysis is the most implemented approach applied for the SHM of bridges (18 studies out of 46) as shown in Table 7. These studies deploy mainly the accelerometer that is capable of assessing the response of the bridge structure due to an excitation force. The static response analysis approaches have been applied in four selected studies. Both dynamic and static response analyses have been implemented together in 15 studies.

## 6. Discussion and Limitations

This section provides a brief discussion of the obtained results. Subsequently, certain limitations of the conducted research have been highlighted.

**Discussion on WSN platforms:** An analytical comparison of WSN platforms in terms of various performance attributes has been presented in Table 8. The attributes of the comparison include the specifications of the data acquisition subsystem (ADC channel as well as resolution), the specifications of the embedded processor (embedded processor as well as data memory) in the target platforms, and the specifications of the wireless channel (radio transceiver, frequency band, and data rate/outdoor range) in the data communication subsystem. From the results in Table 8, it can be observed that most of the WSN platforms deploy ADC with 12-bit resolution. It is adequate to digitize input analog signals from the sensors with high accuracy. In addition, most of the platforms have eight channels in the data acquisition subsystem. These channels are sufficient to acquire input data from various sensors. Regarding the embedded computing subsystem specifications, the ATmega128 (64 KB SRAM + 48 KB Flash) and the TI MSP430F1611 processors (10 KB SRAM + 48 KB Flash) are the most widely used microcontrollers in recent studies with an adequate size of data and program memory for SHM applications. Regarding the specifications of the wireless channel systems, the CC2420 is the most frequently used RF transceiver. The Zigbee technology is the most frequently used wireless protocol in the selected WSN platforms. Finally, Table 9 presents a summary of the accuracy of WSN systems. It is related to the measurement of different quantities such as acceleration and strain, acoustic emission, and impedance. Only 13 studies (out of 37 selected studies) are included as the accuracy of the proposed system is not mentioned in the other 24 selected studies. From Table 9, it can be argued that most of the proposed WSN systems exhibit reasonable accuracy in measuring different quantities compared to wired or commercial systems.

**Discussion on energy harvesting techniques:** This SLR covers the following techniques: vibration, solar, wind, radio frequency (RF), and thermal. The eleven selected studies for vibration energy harvesting techniques are further categorized into six studies for the electromagnetic-based vibration energy harvesting systems and five studies for the piezoelectric material-based vibration energy harvesting systems. Accordingly, a comprehensive analytical comparison of energy harvesting techniques in terms of various performance attributes has been presented in Table 10, Table 11, Table 12, Table 13, Table 14 and Table 15. The main attributes of the comparison process include the output voltage and power, the rectification process, and the integration process. From the results of this SLR, it can be argued that the output power in electromagnetic-based vibration energy harvesting systems is directly proportional to the volume of the electromagnetic. Furthermore, the output power and voltage in solar energy harvesting systems are directly proportional to the size of the PV module. In wind energy harvesting systems, the output power and voltage are directly proportional to the diameter of the wind turbine.

**Discussion on sensor type:** One of the most important considerations when designing an SHM system is the selection of sensors and sensed parameters. Factors such as sensor power consumption and sensed parameters influence the overall network design by influencing routing protocol selection, damage detection algorithm selection, damage localization algorithm selection, and network lifespan. As can be seen in Table 5, the most frequent sensors utilized in the selected studies of this SLR are accelerometers (27 studies), temperature sensors (14 studies), and strain gauges (11 studies).

**Discussion on inspection scale:** The inspection scale of the SHM system for bridges can be classified into two main categories: local and global approaches. As can be seen in Table 6, the most frequent system utilized in the selected studies of this SLR is the global system (31 studies), whereas only 5 studies apply local inspection systems.

**Discussion on response type:** The damage identification process can be classified, in terms of response type, into dynamic (frequencies, mode shapes, or modal damping) and static (strain or stress) systems. As can be seen in Table 7, the most frequent system utilized in the selected studies of this SLR is the dynamic response system (18 studies). On the other hand, only four studies apply static response systems. Both dynamic and static response analysis have been combined in 15 studies of this SLR.

**Limitations of research:** Despite the fact that SLR is purely based on the standard guidelines of [32], there are various small issues that have been highlighted as follows:

- We have used the related keywords during the search process to obtain a significant number of research articles. The obtained research articles have been scanned in a systematic way according to a predefined selection/rejection criterion. Nevertheless, there is no guarantee of the completeness of this scanning process. Moreover, a considerable number of studies have been excluded on the basis of their title. Now, it is quite possible that the titles of the article do not depict the complete research idea of the article. Based on these facts, it is very hard to claim the exhaustiveness of the research conducted in this article.
- In addition to the limitations of the search process and selection/rejection criterion, another probable limitation is the selection of target databases. We have targeted seven well-known databases i.e., IEEE, ELSEIVER, SPRINGER, SAGE, Wiley, MDPI, and Taylor & Francis. These seven databases provide a variety of well-reputed scientific journals and conference proceedings. Nevertheless, there exist several other databases that also provide a lot of scientific articles. Consequently, there is a fair possibility that we have excluded recent research from other databases. However, we firmly believe that the final results of this SLR are not considerably affected because high-quality recent research is available in the selected scientific databases.

## 7. Conclusions

This article has explored WSN platforms and energy harvesting techniques in the context of the SHM process for bridges using an SLR process. As a result, 46 research articles have been selected. The selected articles are classified into three main categories. These categories are WSN platforms, energy harvesting techniques, and a combination of both. Consequently, various WSN platforms have been explored in terms of certain performance parameters. Furthermore, energy harvesting techniques are classified into vibration energy (PZT as well as electromagnetic), solar energy, wind energy, RF energy, and thermal energy. Moreover, the selected studies have been analyzed in terms of some additional design considerations such as the inspection scale (local as well as global) and the response type (static as well as dynamic).

According to the obtained results in this SLR, it can be claimed that vibration-based and solar energy are the most frequently used harvesting techniques for the SHM of bridges. Similarly, accelerometers, strain gauges, and temperature sensors are commonly deployed sensors. In addition, the global inspection scale techniques and the dynamic response analysis method are commonly practiced. Furthermore, it can be argued that the accuracy of WSN platforms in measuring different quantities is comparable with that of the wired systems. Even though there are some limitations (such as the search process, selection/rejection criterion, and the selected databases), it facilitates the selection of appropriate WSN platforms and energy harvesting techniques according to the SHM system requirements.

The energy harvesting techniques considered in this SLR present an alternative power supply for WSN nodes. Nevertheless, they are not sufficient to power the WSN nodes. Therefore, further investigations of novel combined energy harvesting techniques should be considered in future research studies to overcome these limitations. Furthermore, most of the recent studies in the field focus only on the investigations of global damage detection scale methods. Consequently, future research studies should further investigate the implementation of local scale damage detection techniques to localize and assess the impact of damage on bridge structures. This may increase the efficiency of SHM systems in damage detection and increase the lifetime of the bridges.

## Figures and Tables

**Table 1:** State-of-the-art review articles on WSN platforms and EH techniques for the SHM of bridges.

Ref.	Year	Focus	Limitations
[24]	2017	Investigates various methods and techniques in the context of ambient energy sources and EH for SHM applications.	Conducted without SLR guidelines. Not focused on bridge applications. Limited to 2016.
[25]	2023	Reviews the deployment of machine learning algorithms and feature extraction techniques in the SHM process for bridges.	WSN was not explored. EH techniques, inspection scales, and response types were not explored.
[26]	2013	Provides an overview of EH techniques for SHM systems.	It is not systematic and is almost 10 years old. Not focused on bridge applications. RF and thermal techniques were not explored.
[27]	2017	Explores the deployment of WSN platforms for the SHM. The particular focus is made on the damage detection algorithms. Another salient feature is highlighting the network challenges.	Conducted without SLR guidelines. Not focused on bridge applications. Inspection scale and response type were not explored.
[28]	2019	Summarizes the opinion of experts for the deployment of WSN platforms as well as sensor nodes using wires in the context of SHM. The comparison is made for platform, architecture, functionality, communication, and operating system.	Conducted without SLR guidelines. Not focused on bridge applications. Inspection scale was not explored. Response type was not explored.
[29]	2015	Highlights the technologies and developments in the field of SHM using WSNs in bridge engineering.	Conducted without SLR guidelines and is almost 8 years old. Inspection scale and response types were not explored.
[30]	2013	Reviews the WSN technology for the SHM of bridges. Various sensor types have been explored.	Conducted without SLR guidelines and is almost 10 years old. EH techniques, inspection scales, and response types were not explored.
[31]	2022	Describes the methods and challenges for damage identification in bridges. The particular focus is made on the data transmission and analysis for an early damage prediction.	Conducted without SLR guidelines. EH techniques were not compared. Inspection scale was not explored. Response type was not explored.

**Table 2:** A quantitative summary of the search process.

Serial No.	Search Terms	Operator	No. of Search Results						
			IEEE	Springer	Elsevier	SAGE	Wiley	MDPI	Taylor and Francis
1	‘Bridges’ ‘SHM’ ‘WSN’	AND	34	252	171	52	38	96	27
		OR	43,730	44,663	53,187	2446	34,669	91,732	31,689
2	‘Bridges’ ‘SHM’ ‘energy harvesting’	AND	8	40	276	26	47	133	38
		OR	43,014	8251	63,791	218	32,487	94,200	43,969
3	‘Bridges’ ‘solar’	AND	172	2143	95	109	6071	8687	4269
		OR	99,245	56,065	32,451	2557	74,032	158,234	61,236
4	‘Bridges’ ‘wind’	AND	271	6508	137	426	6995	11,103	3719
		OR	98,198	74,122	42,910	3594	68,996	161,536	63,476
5	‘Bridges’ ‘thermal’	AND	573	10,768	78	457	7315	23,598	4279
		OR	97,877	148,310	73,401	6251	53,679	279,510	47,296
6	‘Bridges’ ‘RF’	AND	276	2300	127	78	4428	4403	3145
		OR	88,981	62,249	36,512	2.523	31,984	279,510	32,459
7	‘Bridges’ ‘PZT vibration’	AND	8	365	294	60	2754	13	2911
		OR	21,148	13,233	11,036	860	21,398	84,993	18,398
8	‘Bridges’ ‘Electromagnetic vibration’	AND	33	1282	108	119	4189	113	4287
		OR	23,997	18,247	6043	1090	26,193	85,293	22,218

**Table 3:** Details of data extraction and synthesis.

S. No.	Description	Details
1	Bibliographic information	Title of the research, list of authors, year of publication, and information about the scientific database
**Extraction of data**		
2	Overview	The fundamental idea and goals of the selected research
3	Results	Results achieved by the selected research
4	Validation	The employed testing method for the verification
**Synthesis of data**		
5	WSN platforms	First predefined category
6	Energy harvesting techniques	Second predefined category
7	Sensor types	The type of sensor deployed in the SHM system
8	Inspection scale	The scale of the SHM system (local or global)
9	Response type	The type of the system response (static or dynamic)

**Table 4:** Statistics for energy harvesting techniques.

S. No	Category	Number of Research	Reference
1	Electromagnetic vibration-based	6	[39,42,45,47,67,70]
2	PZT vibration-based	5	[73,74,75,76,77]
3	Solar	6	[38,48,53,68,69,78]
4	Wind	3	[38,69,70]
5	RF	1	[72]
6	Thermal	1	[73]

**Table 5:** Statistics for the utilization of sensors.

S. No	Category	Number of Research	Reference
1	Accelerometer	27	[33,34,35,36,37,41,42,43,46,48,49,50,51,52,53,54,55,56,59,60,61,62,63,64,65,66,78]
2	Temperature sensor	14	[34,36,38,42,46,48,53,56,57,58,60,61,65,66]
3	Strain gauge	11	[36,38,41,46,48,56,60,61,63,66]
4	Humidity sensor	4	[34,57,58,65]
5	Piezoelectric Zirconate-Titanate (PZT) transducer	5	[40,44,57,68,73]
6	Ultrasonic sensor	2	[45]
7	Peak displacement sensor (capacitance-based)	2	[40,71]
8	Acoustic emission sensor	1	[39]
9	Wind gauge	1	[56]
10	RFID	1	[68]
11	Infrared sensor	1	[34]
12	Light sensor	1	[34]
13	Sound sensor	1	[34]
14	PVDF sensor	1	[64]

**Table 6:** Inspection scale classification in terms of global and local methods.

S. No	Category	Number of Research	References
1	Global	31	[33,34,35,36,37,38,40,41,42,43,46,48,49,50,51,52,53,54,55,56,58,60,61,62,63,64,65,66,71,78]
2	Local	5	[39,44,45,57,73]
3	Local and Global	1	[68]

**Table 7:** Statistics for the response type in terms of dynamic and static methods.

S. No	Category	Number of Research	References
1	Static	4	[38,39,58,71]
2	Dynamic	18	[33,35,37,43,44,45,49,50,51,52,54,55,59,62,64,68,73,78]
3	Static and dynamic	15	[34,36,40,41,42,46,48,53,56,57,60,61,63,65,66]

**Table 8:** WSN platforms for the SHM of bridges.

Ref.	Prototype Name	Data Acquisition Specifications		Embedded Computing Specifications		Wireless Channel Specifications			
		ADC Channel	ADC Resolution	Embedded Processor	Data Memory	Radio	Frequency Band	Protocol	Data Rate O/R Outdoor Range
[33]	Xnode	8	24-bit	NXP LPC4357	32 MB SDRAM	Atmel AT86RF23	2.4 GHz IEEE802.15.4, 6LoWPAN	Zigbee	250 Kbps O/R = 1 km
[34]	ODCWSN	6	12-bit	TI MSP430	4 + 32 KB Flash	CC1101	920 MHz	-	250 Kbps
[35]	Prototype	8	12-bit	TI MSP430F5438	16 KB RAM + 256 KB Flash	CC1101	433 MHz	-	500 Kbps O/R = 500–800 m
[36]	S-Mote	-	12-bit	TI MSP430F1611	10 KB SRAM + 48 KB Flash	CC2420	2.4 GHz IEEE802.15.4	-	250 Kbps
[37]	-	4	24-bits	ARM90	40 KB SRAM + 512 KB Flash	-	IEEE802.11	Wi-Fi	-
[38]	GENESI Node v1.0	12	10-bit	TI MSP430F2274	1 KB RAM + 32 KB Flash	CC2420	2.4 GHz IEEE802.15.4	-	250 Kbps O/R = 75 m
[39]	AEPod	8	12-bit	Atmel ATmega1281	8 KB	Atmel AT86RF23	2.4 GHz IEEE 802.15.4	-	250 Kbps
[40]	Thinner	8	10-bit	ATmega128 L	4 KB SRAM + 128 KB Flash	XBee	2.4 GHz IEEE802.15.4	Zigbee	250 Kbps O/R = 100 m
[41]	WISAN	12	12-bit	TI MSP430F1611	10 SRAM + 48 KB Flash	CC2420	2.4 GHz IEEE802.15.4	-	250 Kbps O/R = 75 m
[42]	Self-powered	-	10-bit	TI MSP430F2xxx series	128 B + 2 KB Flash	Nordic nRF24L01	2.4 GHz IEEE802.15.4	-	2 Mbps
[43]	PCB	4	16-bit	Atmel Atmega128	4 KB SRAM + 128 KB	MaxStream 9XCite	900 MHz	-	38.4 Kbps O/R = 300 m
[44]	-	8	10-bit	ATmega644PA	16 Kbyte SRAM, 128 Kbytes Flash memory	Xbee	2.4 GHz IEEE802.15.4	Zigbee	250 Kbps O/R = 100 m
[45]	-	-	12-bit	STM32F-405RG	SRAM	CC2538	-	-	-
[46]	Xnode	8	24-bit	NXP LPC4357	32 MB SDRAM	Atmel AT86RF23	2.4 GHz IEEE802.15.4, 6LoWPAN	Zigbee	250 Kbps O/R = 1 km
[48]	Xnode	8	24-bit	NXP LPC4357	32 MB SDRAM	Atmel AT86RF23	2.4 GHz IEEE802.15.4, 6LoWPAN	Zigbee	250 Kbps O/R = 1 km
[49]	LARA	-	-	Arduino and a Raspberry Pi	-	USB dongle	4G LTE	-	-
[50]	-	24	12-bit	ARM STM32L152	16 Kbytes SRAM + 128 Kbyes Flash	CC1101	-	-	-
[51]	-	-	-	TZ1041	288 Kbtes RAM + 1000 Kbytes Flash	CC1310	sub-GHz	-	-
[52]	-	-	12-bit	ARM M4 STM32	-	Fanstel Bluetooth Low Energy 5.0	-	Bluetooth low energy	200 m
[53]	Windnode	16	-	DSPIC33EP512GP806-I/PT	16 Kbtytes RAM + 512 Kbytes Flash	BT840F	2.4 GHz	Bluetooth low energy	200 m
[54]	Xnode	8	24-bit	NXP LPC4357	32 MB SDRAM	Atmel AT86RF23	2.4 GHz IEEE802.15.4, 6LoWPAN	Zigbee	250 Kbps O/R = 1 km
[55]	-	8	12-bit	EFM32G222F128-QFP48	16 KB RAM + 128 KB Flash	CC1101	868 MHz	-	-
[56]	-	8	10-bit	Atmel atmega128	4 KB SRAM + 128 KB Flash	-	2.4 GHz IEEE802.15.4	Zigbee	-
[57]	-	8		ARM Cortex-M3	20 KB SRAM + 128 KB Flash	CC1310	2.4 GHz IEEE802.15.4, 802.11	-	50 kbps
[58]	CMWX1ZZABZ-091	24	12-bit	STM32L	16 KB SRAM + 128 KB Flash	SX1276	868 MHz	LoRaWAN	250 bps
[59]	WMCIS	8	24-bit	MSP430F5438	16 KB SRAM + 256 KB Flash	CC2520	2.4 GHz IEEE802.15.4	Zigbee	-
[60]	-	-	12-bit	MSP430F1611	10 SRAM + 48 KB Flash	CC2420	2.4 GHz IEEE802.15.4	-	250 bps
[61]	-	-	12-bit	MSP430F1611	10 SRAM + 48 KB Flash	CC2420	2.4 GHz IEEE802.15.4	-	250 bps
[62]	LARA	-	-	Arduino and a Raspberry Pi	-	USB dongle	4G LTE	-	-
[63]	Xnode	8	24-bit	NXP LPC4357	32 MB SDRAM	Atmel AT86RF23	2.4 GHz IEEE802.15.4, 6LoWPAN	Zigbee	250 Kbps O/R = 1 km
[64]	Narada	4	16-bit	Atmel ATmega128	4 KB SRAM + 128 KB Flash additional 128 KB of RAM	CC2420	2.4 GHz IEEE802.15.4	-	-
[65]	ISMO-2	-	12-bit	TI MSP430F1611	10 SRAM + 48 KB Flash	CC2420	2.4 GHz IEEE802.15.4	-	250 bps
[66]	-	-	12-bit	MSP430F1611	10 SRAM + 48 KB Flash	CC2420	2.4 GHz IEEE802.15.4	-	250 bps
[68]	SHiMmer	-	12-bit	Atmel ATmega128	64 KB SRAM + 48 KB Flash	CC1100	433 MHz	-	-
[71]	THINNER	8	10-bit	Atmel ATmega128	64 KB SRAM + 48 KB Flash	XBee	2.4 GHz IEEE802.15.4	Zigbee	250 Kbps O/R = 100 m
[73]	-	-	12-bit	Atmel ATmega128	64 KB SRAM + 48 KB Flash	Atmel AT86RF230	2.4 GHz IEEE802.15.4	-	-
[78]	Imote2	4	16-bit	PXA271 XScale	256 KB SRAM + 32 KB Flash	CC2420	2.4 GHz IEEE802.15.4	-	250 Kbps O/R = 150 m

**Table 9:** The accuracy of proposed WSN systems.

Ref.	Bridge Type	Quantity	Accuracy
[35]	-	Acceleration	The relative error between wired and wireless system natural frequencies estimation is 1.85%.
[37]	Concrete box-girder	Acceleration	The Modal Assurance Criterion (MAC) coefficient is around 0.9 for different modes in modal analysis
[39]	Railway	Acoustic emission	The intensity difference between wired and wireless systems in event detection is 4 dB.
[43]	Concrete box-girder	Acceleration	The error of the acceleration time history collected by the proposed wireless SHM system is 1.1%.
[49]	Footbridge	Acceleration	The system can locate signals up to 0.1 Hz with a 0.5% error.
[51]	Steel-truss	Acceleration	The percentage discrepancies between the bridge frequencies obtained from the proposed wireless system and the two previous systems are below 5–6%.
[52]	Steel-truss	Acceleration	The relative error between the wired and the proposed wireless system in natural frequency estimation is 4.7%.
[54]	Cable-stayed	Acceleration	The measured tension force results using the proposed wireless system matched with the theoretical results with about 0.53% difference; it is also close to the results presented by previous systems with a difference of less than 0.53%.
[56]	Cable-stayed	Acceleration	On average, the difference between the existing wired and the proposed wireless system in cable tension measurement is less than 5 tons f/m.
[61]	Steel- girder	Strain	A ± 0.1 microstrain difference in the strain reading produces less than a 2% absolute difference from the calculated DF based on the acquired strains.
[62]	Steel-girder	Acceleration	The Modal Assurance Criterion (MAC) has been used to compare the mode shapes of the analytical analysis with the experimental study, with MAC values being within the range of 0.93 to 1.00. In addition, comparing the measured eigenfrequencies of the proposed system with those of commercial acceleration accelerometers presents a maximum difference of 0.87 Hz with a difference of 3.3%.
[65]	Wooden model	Acceleration	The absolute average relative difference between the wired and the proposed wireless system for the identified natural frequencies was 0.422%, and the average modal assurance criterion (MAC) value was 0.943.
[78]	Cable-stayed	Acceleration	The difference between the numerical and the experimental frequency values is 0.7%

**Table 10:** Electromagnetic-based vibration energy harvesting systems.

Ref.	Peak Voltage (V)	Volume (cm^3^)	Frequency (Hz)	Acceleration (m/s^2^)		Power (mW)		Rectification	Integration (Storage Capacitor)
				Peak	RMS	Peak	Average		
[39]	-	-	6.4	-	-	-	-	-	rechargeable (Li-Ion) battery
[42]	10	-	3.1	3.79	2.68	12	-	-	-
[45]	2.27	686.9	3.05	1.31	0.18	2.229	0.1147	AC-DC and Boost-up converters	Battery (Stick-and-detect)
[47]	2	68	2	-	0.34	0.0125	0.00502	AC-DC converter	Capacitor
[67]	-	85	1.9	-	0.0546	-	0.00947	-	-
[70]	1	-	3.6	-	-	0.3545	-	-	-

**Table 11:** PZT-based low-frequency vibration energy harvesting systems.

Ref.	Voltage (V)	Frequency (Hz)	Power (mW)	Rectification	Integration
[73]	3.5	-	400	AC-DC converter	Supercapacitor (0.1 F)
[74]	15 p-p	23.2	0.00060	AC-DC converter	-
[75]	1.8	42.2	3.8	AC-DC converter and boost converter	Capacitor (100 µF)
[76]	9.6	40	0.2	-	-
[77]	0.81	7.8	-	-	-

**Table 12:** Solar energy harvesting systems.

Ref.	Voltage (V)	PV Module Size (cm^2^)	Performance		Rectification	Integration
			Max. Power (mW)	Efficiency (%)		
[38]	5	112	450	82 (rechargeable battery) 75 (supercapacitors)	DC-DC converter	Rechargeable (Li-Ion) battery and 50 F supercapacitors
[48]	4	-	3000	-	-	Rechargeable battery
[53]	5.5	38.5	500	-	DC-DC converter	Rechargeable battery
[68]	3.3	100	360	96	Voltage regulators and Boost-up converters	250 F supercapacitors
[69]	29	140	28,000.4	-	Boost-up converters	Battery
[78]	3.7	78	3000	-	-	Rechargeable (Li-poly) battery

**Table 13:** **Wind energy harvesting systems**.

Ref.	Voltage (V)	Wind Turbine Diameter (m)	Performance		Rectification	Integration
			Max. Power (mW)	Efficiency (%)		
[38]	5	0.063	10	85	DC-DC converter	50 F supercapacitors
[69]	29	1.14	22,000.4	-	Boost-up converter	Battery
[70]	64 m	-	0.00809	-	-	-

**Table 14:** RF energy harvesting systems.

Ref.	Voltage (V)	Sensitivity (dBm)	Performance		Rectification	Integration
			Max. Power	Efficiency (%)		
[72]	2.5	−39	13 µJ	60	AC-DC converter and boost converter	Capacitor (100 µF)

**Table 15:** Thermal energy harvesting systems.

Ref.	Voltage (V)	Max. Temp. (°C)	Performance		Rectification	Integration
			Max. Power (mW)	Efficiency (%)		
[73]	1.18	33	400	-	AC-DC Converter	Supercapacitor (0.1 F)

## Data Availability

Not applicable.
